# Persulfidation of Nitrate Reductase 2 Is Involved in l-Cysteine Desulfhydrase-Regulated Rice Drought Tolerance

**DOI:** 10.3390/ijms222212119

**Published:** 2021-11-09

**Authors:** Heng Zhou, Yin Zhou, Feng Zhang, Wenxue Guan, Ye Su, Xingxing Yuan, Yanjie Xie

**Affiliations:** 1 Laboratory Center of Life Sciences, College of Life Sciences, Nanjing Agricultural University, Nanjing 210095, China; hengzhou@njau.edu.cn (H.Z.); 2020116101@njau.edu.cn (Y.Z.); 2018116103@njau.edu.cn (F.Z.); 2016116114@njau.edu.cn (W.G.); 2017116117@njau.edu.cn (Y.S.); 2 Institute of Industrial Crops, Jiangsu Academy of Agricultural Sciences, Nanjing 210014, China; yxx@jaas.ac.cn

**Keywords:** hydrogen sulfide, persulfidation, drought stress, nitrate reductase, l-cysteine desulfhydrase

## Abstract

Hydrogen sulfide (H_2_S) is an important signaling molecule that regulates diverse cellular signaling pathways through persulfidation. Our previous study revealed that H_2_S is involved in the improvement of rice drought tolerance. However, the corresponding enzymatic sources of H_2_S and its regulatory mechanism in response to drought stress are not clear. Here, we cloned and characterized a putative *l-cysteine desulfhydrase* (*LCD*) gene in rice, which encodes a protein possessing H_2_S-producing activity and was named *OsLCD1*. Overexpression of *OsLCD1* results in enhanced H_2_S production, persulfidation of total soluble protein, and confers rice drought tolerance. Further, we found that nitrate reductase (NR) activity was decreased under drought stress, and the inhibition of NR activity was controlled by endogenous H_2_S production. Persulfidation of NIA2, an NR isoform responsible for the main NR activity, led to a decrease in total NR activity in rice. Furthermore, drought stress-triggered inhibition of NR activity and persulfidation of NIA2 was intensified in the *OsLCD1* overexpression line. Phenotypical and molecular analysis revealed that mutation of *NIA2* enhanced rice drought tolerance by activating the expression of genes encoding antioxidant enzymes and ABA-responsive genes. Taken together, our results showed the role of *OsLCD1* in modulating H_2_S production and provided insight into H_2_S-regulated persulfidation of NIA2 in the control of rice drought stress.

## 1. Introduction

Drought is the most widespread and damaging of all environmental stresses, restricting global crop production and food security [1]. Plants can mitigate the effects of drought through the collaboration of complex signal networks. It is well documented that maintaining redox homeostasis and activating ABA signaling could improve plant drought stress tolerance [2,3]. Hydrogen sulfide (H_2_S) has been recognized as a newly gaseous signaling molecule in both animals and plants [4,5]. During the past decades, numerous studies have suggested that H_2_S is involved in various developmental and stress response processes during the whole lifespan in plants [6,7,8,9]. For example, H_2_S is involved in the improvement of drought tolerance by interacting with abscisic acid (ABA) and ion fluxes, thus regulating stomal movement and downstream genes expression in *Arabidopsis* [10,11]. Pretreatment with exogenous NaHS (a H_2_S donor) alleviates drought stress responses by increasing ABA levels through the expression of ABA synthesis genes in wheat and rice [12,13]. Although those studies have demonstrated that H_2_S is involved in regulating many metabolic processes or improving plant tolerance to abiotic stresses, they mainly rely on exogenous application of H_2_S donors, scavengers, and inhibitors to manipulate endogenous H_2_S content [13,14].

In plants, cysteine desulfhydrases (CDes) are one of the most important clusters of H_2_S-producing enzymes catalyzing the degradation of cysteine into H_2_S, pyruvate, and ammonium [15]. There are two types of CDes in plants: l-cysteine desulfhydrase (LCD) and d-cysteine desulfhydrase (DCD) with l-cysteine (l-Cys) or d-cysteine (d-Cys) as substrate, respectively [16]. H_2_S can also be generated as a side reaction of cysteine biosynthesis catalyzed by serine acetyltransferase (SAT) and O-acetyl-serine(thiol)lyase (OAS-TL) [17,18]. Interestingly, an OAS-TL isoform CYSTEINE SYNTHASE (CS)-LIKE protein (CS-LIKE) has been reported that actually catalyzes the desulfuration of l-Cys to H_2_S plus ammonia and pyruvate [18]. Thus, CS-LIKE is a novel l-cysteine desulfhydrase and has been designated as *DES1*. In *Arabidopsis*, *LCD* and *DES1*-mediated endogenous H_2_S production has been widely reported as an important role in facilitating tolerance to various environmental stimuli, including heavy metal and drought stress [11,18,19,20]. However, to date, little information is available about the LCD in rice. A recent study revealed that a putative rice l-cysteine desulfhydrase *LCD* actually encodes a true l-cysteine synthetase [21], suggesting the enzymatic sources of endogenous H_2_S production still need to be further explored.

Signaling by H_2_S is proposed to occur via persulfidation, the oxidative post-translational modification of protein Cys residues (R-SHs) by covalent addition of thiol groups to form persulfides (R-SSHs) [9,22]. Persulfidation modulates protein functions by affecting its biochemical activity and subcellular distribution, thus providing a robust and flexible mechanism for biological regulation in response to metabolic stimuli and environmental cues [23,24]. Recently, by using a comparative and label-free quantitative proteomic analysis approach, almost 13% of the entire annotated proteome proteins were identified as being persulfidated in *Arabidopsis* [23,25]. These proteins are involved in a wide range of biological functions, regulating important processes such as primary metabolism, plant responses to stresses, growth and development, RNA translation, and protein degradation. In guard cells, a complex interaction of H_2_S-mediated persulfidation and ABA signaling has also been described. In the presence of ABA, l-cysteine desulfhydrase1 (DES1) is activated by H_2_S through persulfidation resulting in a burst of H_2_S in guard cells [26]. The increase in H_2_S, in turn, facilitates the over-accumulation of ROS via persulfidation of the NADPH oxidase RESPIRATORY BURST OXIDASE HOMOLOG D(RBOHD), thereby inducing stomatal closure [26]. Besides that, H_2_S-induced persulfidation of ABSCISIC INSENSITIVE 4 (ABI4) is involved in the ABA signaling pathway [27]. These results clearly indicated that H_2_S exerts its biological function through precisely persulfidation of target protein in plants. Previously, we found that exogenous application of NaHS could significantly improve rice drought tolerance by reestablishing redox homeostasis and activation of ABA biosynthesis and signaling [13]. However, the underlying regulatory mechanisms of endogenous H_2_S are not clear.

Nitrate reductase (NR) is a key enzyme in plant nitrogen assimilation, which catalyzes the reduction in nitrate to nitrite in plants [28]. NR plays an important role in plant response to a variety of biotic and abiotic stresses [29]. A study in *Arabidopsis* showed the rate of water loss due to water transpiration was significantly slower in *nia1/nia2* double mutant than in wild-type plants, with *nia1/nia2* double mutant showing the higher expression of ABA-responsive genes and drought tolerance [30], demonstrating plant drought tolerance is negatively regulated by *NR* abundance.

The aim of this study is to explore and characterize the enzymatic sources of endogenous H_2_S production and elucidate the underlying mechanism of how H_2_S confers rice drought tolerance. We cloned and characterized the function of a true *LCD* (*OsLCD1*) from rice. The corresponding biochemical characteristics of purified LCD1 proteins showed that this enzyme predominantly processes H_2_S producing activity. We found that overexpression of *OsLCD1* enhanced rice drought tolerance by activating the expression of related genes encoding antioxidant enzymes and ABA-responsive gene. Further, we demonstrated that persulfidation of NIA2, an NR isoform responsible for the main NR activity, led to a decrease in total NR activity, thus controlling the above genes expression. By combining genetic and molecular analysis, we provide evidence here that H_2_S might through, at least partially, persulfidation-mediated inhibition of NR activity to improve rice drought tolerance.

## 2. Results

### 2.1. Cloning and Functional Characterization of the OsLCD

**Figure 1:**
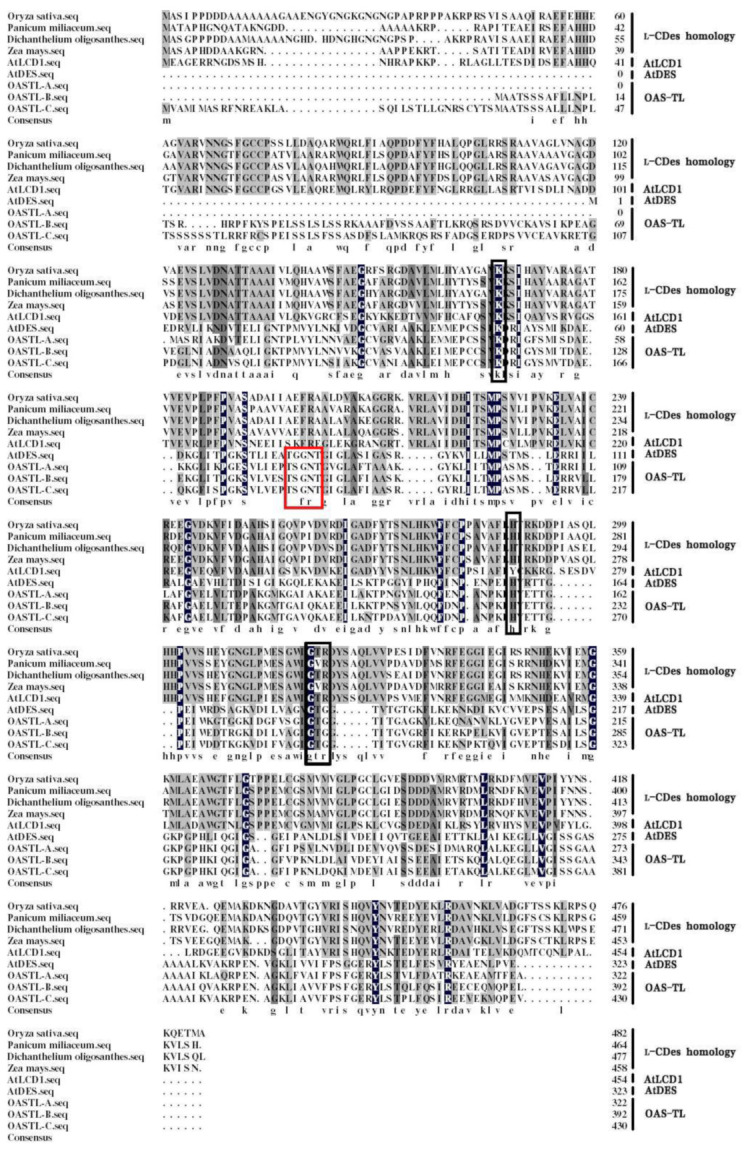
Sequence analysis of CDes, CDes homology, and OAS-TL-A, B, C proteins from the plant. The sequence alignment of L-CDes homology from *Oryza sativa* (XP_015613237), *Panicum miliaceum* (RLN24808), *Dichanthelium oligosanthes* (OEL32418), and *Zea mays* (PWZ10688) and AtLCD1 (NP_001327694), AtDES (OAO92103), OAS-TL-A (AEE83514), OAS-TL-B (AEC10318), and OAS-TL-C (AEE79963) from *Arabidopsis thaliana* was created by DNAMAN with default parameter. Amino acids with blue, black, and gray backgrounds indicate completely or highly conserved residues. The PLP binding sites are shown with a black box, the substrates binding sites of OAS-TL are shown with a red box.

In order to characterize the putative LCD protein in rice plants, the *Arabidopsis LCD* (*At3g62130*) was used as a query sequence to search the homologous gene in *Oryza sativa* by using uniprot-BLAST (https://www.ncbi.nlm.nih.gov/, accessed on 10 July 2019). We found a putative l-cysteine desulfhydrase (*OsLCD1*, *LOC_Os01g18640*) sharing the highest similarity (56%) with *AtLCD*, which encode an OsLCD protein with 482 amino acids residues (Figure 1). The molecular mass of OsLCD1 is 55 kDa, and the theoretical isoelectric point is 5.836 (http://isoelectric.ovh.org, accessed on 20 July 2019). Subsequently, the sequences alignment of the OsLCD1 and CDes homology and OAS-TL family proteins from other species were performed. The results showed that OsLCD1 shares a higher sequence identity with CDes homology from *Panicum miliaceum*, *Dichanthelium oligosanthes*, *Zea mays*, and *Arabidopsis thaliana* in comparison with that of OAS-TL family members from *Arabidopsis thaliana*, including AtDES1. Furthermore, the phylogenetic tree and homology tree were created with MAGE and DNAMAN software with default parameters, respectively. Among those proteins, OsLCD1 is more closely related to the LCD homology proteins from plants (Figure S1).

**Figure 2:**
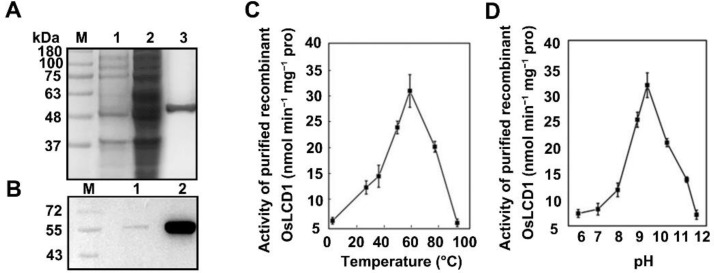
Biochemical characterization of purified recombinant *His*-tagged *OsLCD1* expressed in *E. coli*. (**A**) Expression and purification of OsLCD1 recombinant protein. *OsLCD1* expression was induced with 0.2 mM IPTG for 12 h and then purified by Ni-affinity chromatography. M: Molecular marker; Lane1: Total protein without IPTG induction of *BL21*(*DE3*)//*pET28α-OsLCD1*; Lane2 total protein after IPTG induction of *BL21*(*DE3*)/*pET28α-OsLCD1*; Lane3: Expressed protein purified by Ni-affinity chromatography. (**B**) Western blot of purified OsLCD1 recombinant protein. M: Molecular marker; Lane1: immunoblot of purified protein developed with the polyclonal antiserum against the putative OsLCD1 eluted by NTA-50 buffer; Lane2: immunoblot of purified protein developed with the polyclonal antiserum against the putative OsLCD1 eluted by NTA-50 buffer NTA-100. (**C**,**D**) Temperature and pH dependence of the OsLCD1 reaction. Data shown are means ± SD from three independent measurements.

To validate the biochemical properties of OsLCD1, the corresponding full-length cDNA was cloned, and the recombinant OsLCD1 protein was expressed in *E. coli* as a 6× His N-terminally tagged fusion protein using *pET-28a*(+) vector. The *OsLCD1* fusion protein was purified by nickel affinity chromatography using nickel-nitrilotriacetic acid agarose (Ni-NAT) under non-denaturing conditions to preserve the enzymatic activity. A band appeared in the SDS-PAGE gel at the position corresponding to that of the His-tagged OsLCD1 protein (55 kDa, Figure 2A). The band size and specificity of the OsLCD1 protein were further verified by Western blot analysis using His antibody (Figure 2B). We were able to recover 0.14 mg purified protein per 150 mL of *E. coli* culture with a yield of 36.33% (Table 1). To confirm OsLCD1 functioned as a true LCD, the LCD and OAS-TL activities of both bacterial extracts and purified recombinant OsLCD1 protein were detected, respectively. As shown in Table 1, after purification, the specific LCD or OAS-TL activity (nmol/min/mg pro) of OsLCD1 protein changed from 8.02 or 1900 to 23.93 or 720, with a corresponding purification factor of 2.98 and 0.38. These results suggested that purified OsLCD1 protein might predominately catalyze the degradation of l-cysteine, and the OAS-TL reaction might be a side reaction. This proposition was also reinforced by the results of Km value, showing that the Km for OAS or Na_2_S in the OAS-TL reaction is 25- or 54-fold higher than that for l-cysteine in LCD-catalytic reaction (Table 2), further suggesting a much higher affinity of OsLCD1 for l-cysteine as a substrate. Subsequently, biochemical analysis showed that the optimum temperature range of purified OsLCD1 protein was 50 to 80 °C (Figure 2C). The rate of the LCD reaction increased to its maximum value at a temperature of 60 °C and declined thereafter. Meanwhile, the LCD activity of OsLCD1 under different pH was determined at 60 °C, and results showed that the optimal pH of OsDCD1 was 9.5 (Figure 2D).

### 2.2. Overexpression of OsLCD1 Enhance Endogenous H_2_S Production and Drought Tolerance in Rice

**Figure 3:**
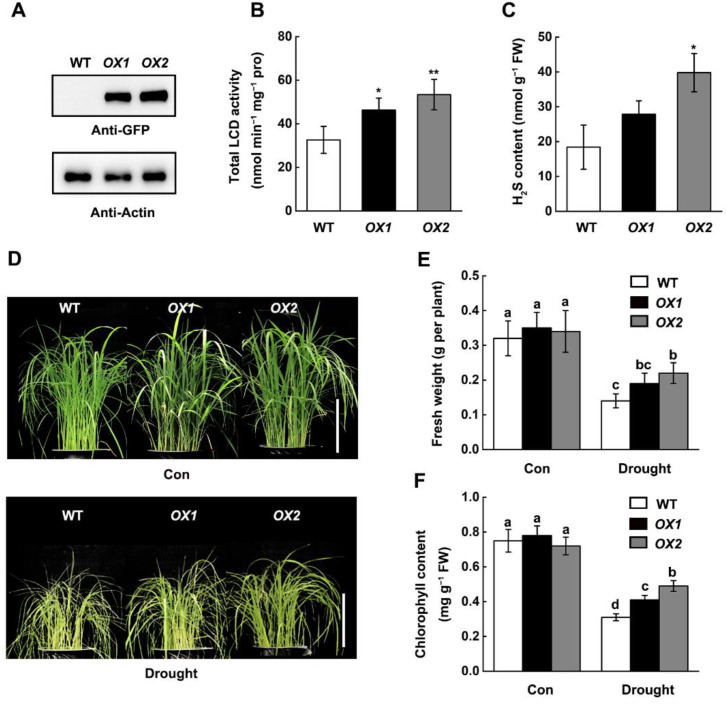
Overexpression of *OsLCD1* enhances rice drought tolerance. (**A**) Identification of *35S:OsLCD1-GFP* overexpression lines. The total protein from two independent overexpression lines (*OX1* and *OX2*) and wild-type (cv. Wuyunjing 7, WT) was extracted and was analyzed by immunoblotting with an anti-GFP antibody. (**B**,**C**) Overexpression of *OsLCD1* increases LCD activity and H_2_S production in 14-day-old rice plants. (**D**) Drought stress tolerance assay. Well-irrigated 14-day-old wild-type, *OX1*, and *OX2* rice seedlings were exposed to drought stress by withholding water for 6 days. Pictures were then taken. Scale bar = 10 cm. (**E**,**F**) The related fresh weight and chlorophyll content were determined. Data are means ± SD (*n* = 3). Statistical comparisons were performed by independent samples *t*-test (two-tailed) between leaves from wild-type and *OsLCD1* overexpression lines (* *p* < 0.05, ** *p* < 0.01). Different lower case letters indicate significant differences at *p* < 0.05 (one-way ANOVA, Duncan’s multiple range tests).

To investigate the physiological role of *OsLCD1* in rice, two independent *35S:OsLCD1-GFP* overexpression lines (*OX1* and *OX2*) were generated by introducing the *pCAMBIA1305-OsLCD1-GFP* expression construct into Wuyunjing 7. Firstly, the overexpression of *OsLCD1* was confirmed by immunoblot analysis. The results showed that the band signal of *OsLCD1-GFP* was detected in protein extracts from both two transgenic lines, but not wild-type (cv. Wuyunjing 7) (Figure 3A). Furthermore, biochemical characterization results revealed that the total LCD activity in *OX1* and *OX2* was increased by 43.7% and 71.8% compared to the wild-type plants (Figure 3B). This result further confirms that the LCD1 protein is a true LCD enzyme. Accordingly, the endogenous H_2_S content in *OX1* and *OX2* was about 47.5% and 102.1% higher than those of wild-type plants (Figure 3C).

Our previous study has shown that exogenous H_2_S could alleviate rice drought stress [13]. We then wonder whether the overexpression of *OsLCD1* would affect rice drought tolerance. Thus, two-week-old rice seedlings (WT, *OX1,* and *OX2*) were subjected to drought stress for 10 days. We observed that overexpression of *OsLCD1* significantly improved the growth performance of rice seedlings under drought stress (Figure 3D). Compared with the wild type, the fresh weight was increased by 29% and 36% (Figure 3E), and the chlorophyll content was increased by 28% and 39% in *OX1* and *OX2* plants (Figure 3F), respectively. These results indicated that overexpression of *OsLCD1* improves rice drought tolerance.

To investigate the molecular mechanism of *OsLCD1* in response to drought stress, the expression profiles of genes involved in drought stress response were determined. RT-qPCR results showed that the transcript of genes encoding antioxidant enzymes, including *ascorbate peroxidase 2* (*APX2*) and *catalase* (*CATA*), and *a basic leucine zipper* (*bZIP*) *transcription factor 23* (*bZIP23*) and *a dehydration responsive element-binding protein* (*DREB*) were increased by drought stress in wild-type plants, while this induction was further enhanced in *OX2* plant (Figure S2). These results indicated that overexpression of *OsLCD1* may improve rice drought tolerance via modulating the expression of genes involved in drought stress response.

**Figure 4:**
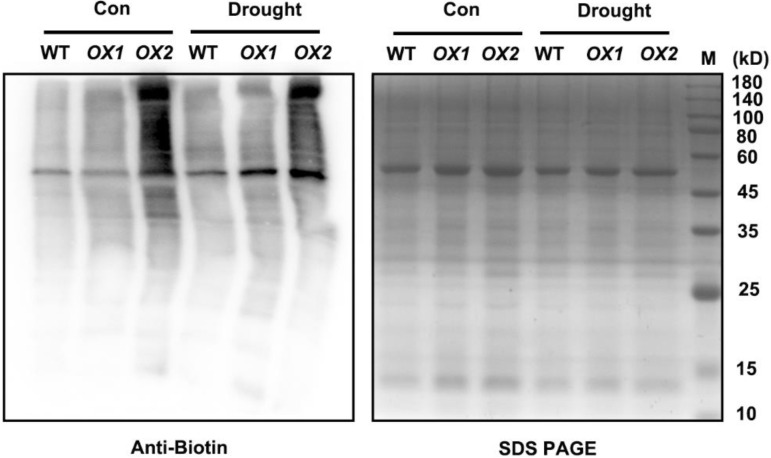
*OsLCD1*-mediated persulfidation of rice total protein. Fourteen-day-old rice seedlings of wild-type (cv. Wuyunjing 7, WT), *OX1*, and *OX2* were isolated from cultivated culture and placed in filter paper for dehydration for 3 h. Afterward, proteins were extracted from 0.2 g of leaves and subjected to the modified biotin switch method, and the labeled proteins were detected using protein blot analysis with antibodies against biotin. Coomassie brilliant blue-stained gels were presented to show that equal amounts of proteins were loaded. Numbers on the right of the panels indicate the position of the protein markers in kDa.

H_2_S-mediated persulfidation has been reported that regulate diverse cellular signaling pathways [9]. To investigate whether overexpression of *OsLCD1* affects the protein persulfidation level in rice seedlings, we determined the persulfidation level of total protein from wild-type *OX1* and *OX2* plants under normal and drought stress conditions. A tag-switch assay in which persulfidated Cys was labeled with cyan-biotin and could specifically be detected by anti-biotin immunoblot analysis was used [23,24]. The immunoblotting results showed that drought stress significantly enhanced the protein persulfidation level in all rice plants, while the protein persulfidation level was higher in *OX1* and *OX2* compared with wild-type (Figure 4). These results indicated that *OsLCD1*-mediated persulfidation may involve in rice drought tolerance.

### 2.3. Dehydration-Triggered Inhibition of NR Activity Was Correlated with Endogenous H_2_S Content

**Figure 5:**
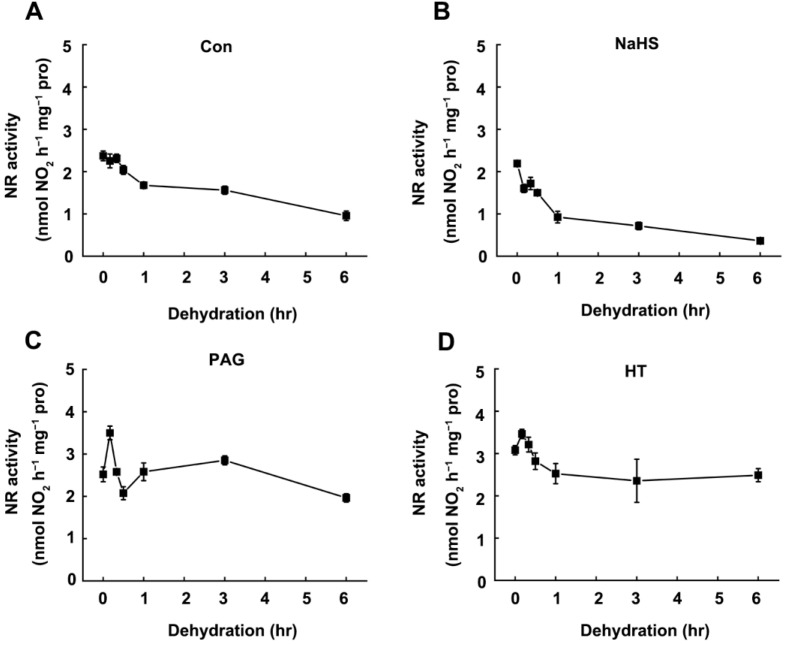
Regulation of NR activity by H_2_S. The 14-day-old rice seedlings cultured under normal conditions (**A**) or retreated with NaHS (100 uM, **B**), PAG (1 mM, **C**), or HT (1 mM **D**). For the dehydration time-course experiment, Leaf blade branches were isolated from cultivated soil and placed in filter paper. The leaves samples were harvested, and the total NR activity was measured at indicated time points. Data are means ± SD (*n* = 3).

Previous studies revealed that NR plays an important role in plant stress response [31]. To investigate whether NR is involved in the endogenous H_2_S-enhanced rice drought tolerance, we detected the changes of NR activity in rice leaves in response to drought stress. In comparison with the control plants, NR activity was decreased in rice seedling leaves after dehydration (Figure 5A). For example, NR activity was rapidly decreased by 33.3% within 1 h after dehydration and unchanged until 3 h, and then further decreased by 67.2% at 6 h. The changes in NR activity showed the opposite tendency with endogenous H_2_S production in response to dehydration stress [13]. Meanwhile, the pretreatment of NaHS reinforced the decrease in NR activity after dehydration, indicating that dehydration-triggered inhibition of NR activity may regulate by endogenous H_2_S (Figure 5B). To further verify this, hypotaurine (HT, a H_2_S scavenger) [32] or dl-propargylglycine (PAG, an l-DES inhibitor) [33] was used. With respect to the rapidly decreased NR activity in response to dehydration stress by exogenous application of NaHS, pretreated with HT or PAG significantly alleviated dehydration-induced inhibition of NR activity (Figure 5C,D). Thus, these results clearly indicated that dehydration-triggered inhibition of NR activity was correlated with endogenous H_2_S content.

**Figure 6:**
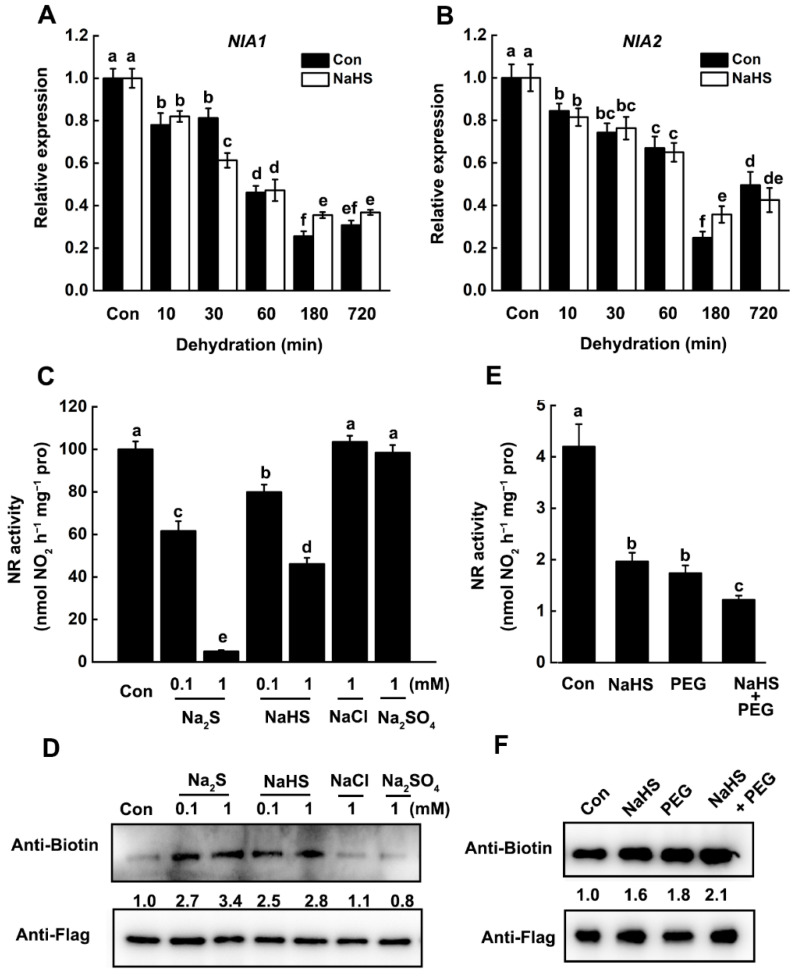
H_2_S-mediated persulfidation inhibits NR activity in response to drought stress. (**A**,**B**) Time-course expression profiles of *NIA1* and *NIA2* in rice seedlings pretreated with or without NaHS followed by dehydration. Fourteen-day-old wild-type (cv. Wuyunjing 7, WT) rice seedlings were pretreated with or without NaHS (100 μM) for 2 h and then placed in filter paper for dehydration treatment to mimic drought stress. Leaves were harvested for RT-qPCR analysis at the indicated time point. Expression levels are relative to corresponding untreated wild-type samples (control) after normalization to reference genes of *OsActin1* and *OsActin2*. (**C**) Persulfidation inhibits NIA2-related NR activity in tobacco. *N. benthamiana* leaves were transiently overexpressed *35S:NIA2-Flag* construct. Total proteins were extracted, and NIA2-Flag protein was harvested by immunoprecipitation with anti-Flag agarose beads. NIA2-Flag protein-related NR activity was detected after being treated with or without different sulfur-containing chemicals for 30 min. (**D**) The persulfidation level of NIA2-Flag protein from (**C**) was analyzed by immunoblotting with an anti-Biotin and anti-Flag antibody. The persulfidation levels are relative to corresponding untreated control samples after normalization to the anti-Flag signal abundance. (**E**) Persulfidation-inhibited NIA2-related NR activity upon osmotic stress in rice. Rice protoplasts of the *nia2* mutant (cv. Dongjin) were transfected with *35S:NIA2-Flag*. After 12 h incubation, protoplasts were treated with or without NaHS (100 μM) in the absence or presence of PEG6000 (10%) for 1 h. Total proteins were extracted for the determination of NR activity. (**F**) The persulfidation level of protein from (**E**) was analyzed by immunoblotting with an anti-Biotin and anti-Flag antibody. The related persulfidation level is relative to corresponding untreated control samples after normalization to the anti-Flag signal abundance. Data are means ± SD (*n* = 3). Lower case letters indicate significant differences at *p* < 0.05 (Duncan’s multiple range tests).

To determine whether the decreased NR activity was caused by transcriptional level regulation or post-translational modification, the expression profiles of genes encoding NR were verified. In rice, there are two NR encoding genes, *NIA1* and *NIA2*. The RT-qPCR result showed that the transcripts level of both *NIA1* and *NIA2* was gradually decreased in rice seedling leaves after dehydration (Figure 6A,B). However, pretreatment of NaHS has no significant effect on the dehydration-inhibited gene expression of *NIA1* and *NIA2*, indicating H_2_S may regulate NR activity at the post-translational level.

### 2.4. H_2_S-Mediated Persulfidation-Inhibited NR Activity

It is plausible that NR activity was regulated by H_2_S-mediated persulfidation. Subsequently, we determine the effects of the exogenous application of H_2_S donors on NR activity. In rice, the transcriptional level of *NIA2* is markedly higher than that of *NIA1*, and *NIA2* mutation causes more than 90% loss in NR activity [34,35]. Thus, we first clone the rice *NIA2* gene and transiently overexpressed *35S:NIA2-Flag* construct in *N. benthamiana* leaves. After 12 h incubation, the NIA2 protein was immunoprecipitated by using an anti-Flag antibody and treated with different H_2_S donors. The results showed that treatment of both well-known H_2_S donors, NaHS and Na_2_S, significantly decreased NR activity (Figure 6C). When 1 mM NaHS was applied, the NR activity was decreased by 55%, while 1 mM Na_2_S caused a 93% loss in NR activity. It should be mentioned that treatment with NaCl or Na_2_SO_4_ fails to reduce the NR activity. These results suggested that H_2_S or HS^−^, rather than other compounds regulates the NR activity. These changes in NR activity were consistent with the corresponding persulfidation level of NIA2, which was enhanced by both H_2_S donors rather than NaCl or Na_2_SO_4_ (Figure 6D).

To determine whether NIA2 could be persulfidated in vivo, the protoplasts from *nia2* rice mutant (cv. Dongjin) with transiently overexpressed *35S:NIA2-Flag* were treated with or without NaHS in the presence or absence of polyethylene glycol (PEG), which further mimic drought stress. As expected, immunoblotting results showed that NIA2 protein was persulfidated in rice protoplasts, and NaHS pretreatment enhanced its persulfidation level (Figure 6E). Importantly, the persulfidation of NIA2 protein was significantly enhanced by PEG treatment, while this could be further strengthened by NaHS. These results on the persulfidation level of NIA2 protein were consistent with the changes in their enzymatic activity (Figure 6F), indicating that dehydration-triggered inhibition of NR activity was controlled by H_2_S-mediated persulfidation.

**Figure 7:**
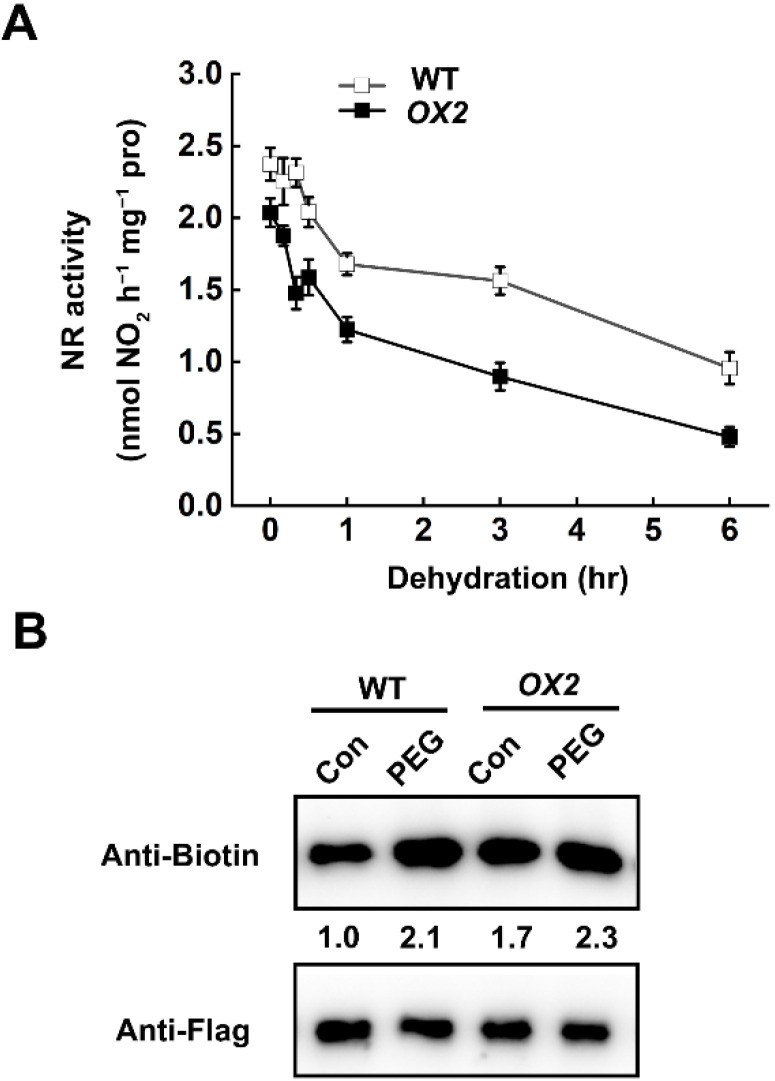
Overexpression of *OsLCD1* enhances persulfidation and the activity decrease in NR. (**A**) Dehydration-inhibited NR activity. The 14-day-old wild-type (cv. Wuyunjing 7, WT) and *OX2* rice seedlings were cultured under normal conditions. For the dehydration time-course experiment, leaf blade branches were isolated from cultivated soil and placed in filter paper. The leaves samples were harvested, and the total NR activity was measured at indicated time points. Data are means ± SD (*n* = 3). (**B**) Wild-type and *OX2* rice protoplasts with transiently expressed *NIA2-Flag* were treated with or without 100 μM NaHS in the absence or presence of 10% PEG6000 1 h, and then the total proteins were extracted and analyzed by immunoblotting with an anti-Biotin and anti-Flag antibody.

In order to validate the contribution of *OsLCD1* in PEG-induced NIA2 persulfidation, we examined the persulfidation level of NIA2 protein in wild-type (cv. Wuyunjing 7, WT) and *35S:OsLCD1-GFP* overexpression rice plants (*OX2*). With this aim in mind, the *35S:NIA2-Flag* construct was separately transiently expressed into protoplasts of wild type and *35S:OsLCD1-GFP* overexpression line (*OX2*). The immunoblotting result showed that PEG treatment induced the persulfidation of NIA2 protein in protoplasts of wild type, while this was further intensified in *the OX2* line (Figure 7A). Moreover, we found that the NR activity was decreased faster in the *OX2* line as compared to wild-type plants upon the dehydration stress (Figure 7B). These results demonstrated that persulfidation-mediated inhibition of NR activity may confer rice drought tolerance.

### 2.5. Knock down of NIA2 Enhances Rice Drought Tolerance

**Figure 8:**
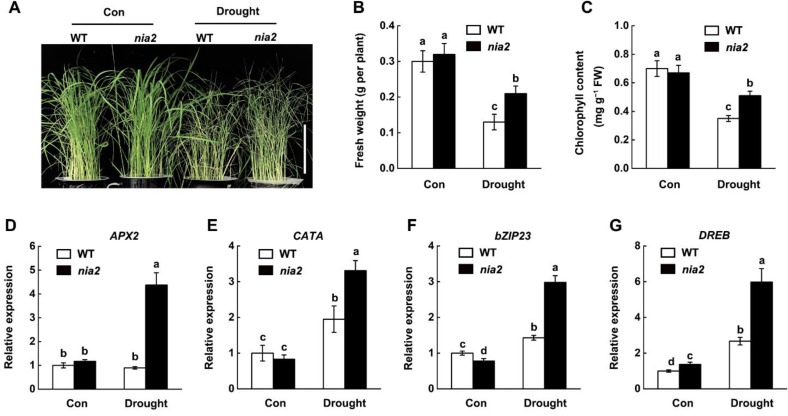
Knockdown of *NIA2* enhances rice drought tolerance. (**A**) Photographs of 14-day-old well-irrigated wild-type (cv. Dongjin, WT) and *nia2* mutant rice seedlings were withdrawn from irrigation for 6 days. Scale bar = 10 cm. (**B**,**C**) The related fresh weight and chlorophyll content were determined. (**D**–**G**) Relative transcript levels of genes involved in drought stress response in rice seedling leaves were quantified after 4 days of drought stress by qRT-PCR. Expression levels are relative to corresponding untreated wild-type samples (control) after normalization to *OsActin1* and *OsActin2*. Data are means ± SD (*n* = 3). Lower case letters indicate significant differences at *p* < 0.05 (Duncan’s multiple range tests).

To investigate the biological role of NR inhibition in rice drought stress response, drought stress tolerance of wild-type and *nia2* mutant was compared. We observed that mutation of *NIA2* significantly improved the growth performance of rice seedlings under drought stress (Figure 8A). Compared with the wild-type (cv. Dongjin, WT), the fresh weight was increased by 36% and 29% (Figure 8B), as the chlorophyll content was increased by 39% in the *nia2* plant (Figure 8C), respectively. These results indicated that mutation of *NIA2* improves rice drought tolerance.

To further investigate the molecular mechanism of *NIA2* in response to drought stress, the expression profiles of genes involved in drought stress response in the *nia2* mutant were determined. RT-qPCR results showed that the transcript of genes encoding antioxidant enzymes, including *APX2* and *CATA*, were significantly higher in *nia2* mutant as compared to wild-type plants upon drought stress (Figure 8D,E). Meanwhile, after drought stress, the induction of *bZIP23* and *DREB* genes in *nia2* mutants was further enhanced compared with the wild type (Figure 8F,G). Based on these findings, we concluded that *NIA2* negatively regulates rice drought tolerance.

## 3. Discussion

### 3.1. A True l-Cysteine Desulfhydrase Confers Rice Drought Tolerance

The importance of cysteine (Cys) in plants is defined not only by its role as an amino acid in primary and secondary metabolisms but also by its function as a metabolic precursor of essential biomolecules [36,37]. In plant cells, H_2_S is generated through enzymatic pathways that are closely related to Cys metabolism [16]. l-cysteine DESULFHYDRASE (LCD) and d-cysteine DESULFHYDRASE (DCD) degrade l/d-Cys to H_2_S, pyruvate, and ammonia and contribute to the production and biological function of H_2_S in the cell [38]. Here, we discovered and characterized a rice LCD encoding gene that shares the highest similarity (56%) with the AtLCD [10,39]. The phylogenetic and homology analysis showed that OsLCD1 is more closely related to the LCD homology proteins from plants rather than the OAS-TL family (Figure 1 and Figure S1).

Up to now, progress has been made in the characterization of the CDes, which usually possess bi-functional activities [9]. For example, the AtDCD1 (*At1G48420*) was identified as a d-CDes and also possessed ACCD activity [40,41]. AtDCD2 (*At3g26115*) catalyzes the release of H_2_S from d-cysteine as well as l-cysteine [16]. Moreover, due to the reversibility of catalytic reactions, those enzymes sometimes exhibit opposite activities. For example, *Arabidopsis* DES1, a member of the OAS-TL family, is involved in l-cysteine degradation rather than biosynthesis [18]. By contrast, LCD2, a rice AtLCD homolog, predominantly exhibits cysteine biosynthesis activity and is, therefore, a true cysteine synthetase [21]. Our results showed that the Km of recombinant OsLCD1 protein for OAS or Na_2_S in the OAS-TL reaction is 25- or 54-fold higher than that for l-cysteine in LCD-catalytic reaction, indicating OsLCD1 predominantly catalyzes the degradation of l-cysteine and thus is a true LCD (Table 2).

The deduction that OsLCD1 is a true LCD was also supported by the analysis of its overexpression rice plants. The total LCD activity in *OX1* and *OX2* was increased by 43.7% and 71.8% compared to the wild-type plants (Figure 3B). Accordingly, the endogenous H_2_S content in *OX1* and *OX2* was about 47.5% and 102.1% higher than those of wild-type plants (Figure 3C). Previous studies reported the involvement of CDes in drought resistance [10,11]. Our result also demonstrated that overexpression of *OsLCD1* improves rice drought tolerance (Figure 3D). As the main source of H_2_S production in plant cells, the biological function of CDes largely relies on H_2_S [19,42,43]. *OsLCD1*-improved rice drought tolerance may derive from the increase in endogenous H_2_S content.

### 3.2. Molecular Mechanisms Underlying the Effects of H_2_S on Drought Tolerance

Numerous biochemical and genetic results have undoubtedly established that the signaling action of H_2_S in cells through persulfidation has important consequences for many physiological processes in plants [22,43,44]. Here, we found that the persulfidation widely exists in rice proteome under normal conditions and was differentially changed by drought stress (Figure 4). This further indicates that persulfidation may involve in rice drought stress response.

Nitrate reductase (NR) is a key enzyme for nitrogen assimilation and acquisition and plays a central role in plant biology and signaling transduction [28,45]. Previous results showed that the NR activity declined rapidly in response to drought stress, indicating NR may act as a negative regulator in plant drought stress response [46]. Most recently, it was reported that suppression of nitrate assimilation by regulating the expression of *NR* under drought stress could contribute to drought tolerance [47]. Similarly, our results showed that the expression of *NR* genes (Figure 6A,B) and related NR activity (Figure 5A) were gradually decreased under drought stress, indicating drought stress-induced inhibition of NR activity may attribute to the transcription regulation. These results were consistent with a previous study in maize leaves, which shows the decrease in maximal extractable NR activity was accompanied by a decrease in NR transcripts [46]. However, we observed that along with the decrease in NR activity, the endogenous H_2_S content was gradually decreased after dehydration stress [13], while pretreatment of NaHS could promote the dehydration stress-induced inhibition of NR activity (Figure 5A,B). It indicated that dehydration-triggered inhibition of NR activity may correlate with endogenous H_2_S content. This deduction was further confirmed by the application of H_2_S scavenger, HT and LCD inhibitor, PAG, which delay or attenuate the inhibition of NR activity under dehydration stress (Figure 5C,D). Moreover, pretreatment of NaHS has no significant effect on the abundance of *NIA1* and *NIA2* (Figure 6B), illustrating that H_2_S-promoted inhibition of NR activity may occur at the post-translational level.

NR is a highly regulated enzyme that is regulated at a variety of levels, including transcriptional-level regulation and post-translational modification in response to various environmental stimuli. For instance, the activity of NR in plants changes rapidly in response to various environmental stimuli, such as nitrate, light, plant hormones, low temperature, and drought stress [46,48,49,50]. Recent study on the interplay of persulfidation and phosphorylation of SnRK2.6 in Arabidopsis stomata regulation and drought tolerance [51] provide a good example for understanding the regulatory mechanism of NR in response to environmental stimuli. Previous studies demonstrated that NR activity was controlled by phosphorylation/dephosphorylation in plant cells. This regulatory model allows the NR transformation between high activity and low activity state [52,53]. Interestingly, both *NIA1* and *NIA2* protein was found in the *Arabidopsis* persulfidation proteome [23], revealing a new regulatory mechanism for NR functions. Here, our study showed that the persulfidation modification was detected in the OsNIA2 protein (Figure 6D,F), which is responsible for more than 90% NR activity in rice [35]. Drought stress significantly induced persulfidation of NIA2 protein, while this could be further enhanced by NaHS pretreatment or overexpression of *OsLCD1* (Figure 6F and Figure 7B). The drought stress or NaHS treatment triggers the persulfidation of NIA2 protein and thus inhibits its activity. These results demonstrated that NR activity was also controlled by H_2_S-mediated persulfidation in response to drought stress. 

Rice seedlings grown in nitrate-deficient conditions are more tolerant to drought stress than of nitrate-sufficient conditions, indicating that decreased nitrogen assimilation contributes to the drought tolerance of rice [47]. This could be a strategy for plants balancing growth and defense responses under stress conditions. Consistently, the loss-of-function mutants of *OsNR1.2* [47] and *nia2* mutant, which both impaired nitrogen assimilation, are more tolerant to drought stress (Figure 8). A zinc finger transcription factor DROUGHT AND SALT TOLERANCE (DST) was specifically responsible for the suppression of *OsNR1.2* expression, but not *OsNIA2* in response to drought stress. As a consequence, *osnr1.2* mutant plants exhibited similar enhanced stomatal closure and drought tolerance as *dst* mutant plants. As the side reaction during NR-catalyzed nitrogen assimilation, the production of NO, an important signaling molecule, also contributed to the biological function of NR [31]. However, Since NO-deficient plants are markedly resistant to water deficit, the reduced water losses in NO-deficient plants may be due to hypersensitivity to ABA, thus leading to NO-independent inhibition of stomata opening and enhanced closure by ABA. In *Arabidopsis*, ABA-mediated regulation of stomata closure may not be necessarily dependent on de novo biosynthesis of NO through any of the proposed NR-mediated pathways [30]. In our study, the enhanced expression of ABA-responsive genes in the nia2 mutant was observed (Figure 8F,G), further confirming the importance of the NO-independent pathway in plants’ response to drought stress.

Thus, the effects of H_2_S-mediated persulfidation on NIA2 suggest a new mechanism for the modification of the NR protein itself in response to drought stress. More importantly, our results indicated that H_2_S regulates signaling pathways in response to drought stress through persulfidation of NR protein, which led to the faster and more efficient inhibition of NR activity than through transcription regulation. These data provide new information that will benefit future studies on NR functional regulation in plants and expand the biological function of gasotransmitter H_2_S.

## 4. Conclusions

In summary, we cloned and characterized a gene encoding an H_2_S-producing enzyme in rice and named *OsLCD1.* Overexpression of OsLCD1 results in enhanced endogenous H_2_S production, persulfidation of total soluble protein, and confers rice drought stress. We further elucidated a key mechanism of OsLCD1/H_2_S-improved rice drought stress. Upon drought stress, H_2_S induces persulfidation of NIA2, an NR isoform responsible for the main NR activity, thus decreasing total NR activity in rice. The inhibition of NIA2 activity improved the drought-responsive genes expression and further led to enhancement of drought tolerance in rice, as proved by the *nia2* mutant analysis. Combined with our previous knowledge of H_2_S beneficial role on plant growth performance under various environmental stresses, our results contribute to the effective use of H_2_S in agriculture, not only by exogenous administration of H_2_S donors but also by genetic manipulation regarding H_2_S metabolic pathways. Moreover, our results shed new light on the understanding of crop genetic improvement strategies through exploring and manipulating the other components that effectively regulate NR activity to balance crop growth/nitrogen assimilation and adaptation to stress.

## 5. Materials and Methods

### 5.1. Plant Materials, Growth Condition, and Treatment

Rice (*Oryza sativa* L., Wuyunjing 7 [54], and Dongjin [35]) was used in this study. Seeds were surface-sterilized and germinated in distilled water for 2 days at 28 °C. For drought stress experiments, germinated seeds were sowed into a 550 mL black opaque plastic beaker with soil in the glasshouse. The soil was taken from a field experiment site in Nanjing Agricultural University in Nanjing, Jiangsu. After two weeks, seedlings were withdrawn for irrigation for 8 days. After treatments, the corresponding phenotypes, including fresh weight and chlorophyll content, were measured.

### 5.2. Sequence Alignment and Phylogenetic Analysis

The alignment and phylogenetic tree of L-CDes homology from *Oryza sativa* (XP_015613237), *Panicum miliaceum* (RLN24808), *Dichanthelium oligosanthes* (OEL32418), and *Zea mays* (PWZ10688) and AtLCD1 (NP_001327694), AtDES (OAO92103), OAS-TL-A (AEE83514), OAS-TL-B (AEC10318), and OAS-TL-C (AEE79963) from *Arabidopsis thaliana* was performed and constructed according to the method described previously [8].

### 5.3. Cloning, Expression, and Purification of Recombinant OsLCD1

Total RNA was extracted from leaves of 14-day-old rice plants using Trizol reagent (Invitrogen, Gaithersburg, MD, USA) according to the manufacturer’s instructions. The reverse transcription reaction was carried out to obtain cDNA by using the Super Script First-Strand Synthesis System for RT-PCR (Transgene, Beijing, China). To obtain the putative *L-CDes1* cDNA from Oryza sativa, the forward primer (5′-ATGGCGTCGATCCCGCCGGAT-3′) and the reverse primer (5′-TCAGGCCATCGTTTCCTGCTTC-3′) were used. The full length of *OsLCD1* was introduced into the *pET-28a(+)* vector at the sides of X*hoI* and B*amHI* using a homologous recombination technique (Vazyme). After that, the recombinant vector was transferred into *E. coli* strain *Rosetta* (*DE3*) for protein expression. Briefly, the freshly inoculated Rosetta strain was grown at 37 °C with vigorous shaking for 4 h, at which point the OD600 of the culture was 0.5~0.6. Then 0.2 mM isopropyl-β-d-thiogalactopyranoside (IPTG) was added and cultivated for 12 h at 16 °C. The purification was performed under non-denaturing conditions by affinity to nickel resin using the Ni-NTA Purification System (Invitrogen) according to the manufacturer’s instructions.

### 5.4. SDS-PAGE of Recombinant OsLCD1 and Western Blotting

Recombinant OsLCD1 protein was purified and then subjected to 12.5% SDS-PAGE. After electrophoresis, the protein was transferred from gel to the polyvinylidene difluoride (PVDF) membrane. The membrane was incubated in phosphate-buffered saline (PBS) with 5% bovine serum albumin (BSA) for 1 h at room temperature. After being washed with PBS/Tween buffer three times, immunoblot analysis was performed with relevant antibodies. The anti-His antibody was used at 1:5000 dilution. A secondary antibody was also used at 1:5000 dilution. The bands were visualized using enhanced chemiluminescence (ECL) reagents (Vazyme).

### 5.5. Enzyme Activity Measurements

The OsLCD1 activity was measured by the release of H_2_S from l-cysteine. The assay contained a total of 3 mL 100 mM Tris/HCl pH 8.5, various amounts of different protein extracts, and 2.5 mM DTT. The reaction was started by the addition of 1 mM l-cysteine, incubated for 30 min at 37 °C, and terminated by adding 300 μL of 30 mM FeCl_3_ dissolved in 1.2 N HCl and 300 μL 20 mM N,N-dimethyl-pphenylenediamine dihydrochloride dissolved in 7.2 N HCl [55,56]. The formation of methylene blue was determined at OD670 nm by using a spectrophotometer. Solutions with different concentrations of Na_2_S were prepared used for the quantification of the enzymatically formed H_2_S. OAS-TL activity was measured using the method described previously [57] in soluble bacterial or purified protein extracts. Nitrate activity was indicated by active nitrate reductase (NRAact). Briefly, the leaf samples or protoplasts were harvested and ground in the extraction buffer containing 25 mM potassium phosphate buffer (pH 8.8) and 10 mM cysteine. The protein extracted in the presence of excess Mg^2+^ is considered to be the NRAact in situ in leaf tissues, while NRAmax is measured in the presence and preincubation of EDTA for 30 min. The reaction mixture contained 0.4 mL of the extracted aliquots, 1.2 mL of a 0.1 mM potassium phosphate buffer (pH 7.5), 0.1 mM KNO_3_, and 0.4 mL of 0.25 mM nicotinamide adenine dinucleotide (NADH). NRA was expressed as μmol NO^2−^g^−1^ FW h^−1^.

### 5.6. Construction and Characterization of OsLCD1 Overexpression Lines

Transgenic lines (*OsLCD1* overexpression lines) were generated by Biorun Biotechnology. To obtain the transgenic plants overexpressing *OsLCD1*, the full-length coding DNA sequence of *OsLCD1* was inserted into the plant binary vector *pCAMBIA1305-GFP*. Then, the *OsLCD1* gene under the control of *CaMV 35S* promoter was transformed into rice (cv. Wuyunjing 7) by the Agrobacterium-mediated transformation method [58]. The progeny was selected by hygromycin and Western blotting with anti-GFP antibodies. Homozygous T3 seeds of the transgenic plants were used for further analysis.

### 5.7. Protoplast Preparation and Transiently Expression of OsNIA2

Stem and sheath tissues from 100 10-day-old rice seedlings were cut into approximately 0.5 mm strips and were used for protoplast isolation [59]. The method and details of *OsNIA2* gene clone and transient expression in rice protoplast were according to a previous study [60]. Briefly, the *1300221-OsNIA2-Flag* plasmid was transfected into 1 mL rice protoplast from WT, *OX2*, or *nia2* plants using a PEG-calcium-mediated method. After 12 h incubation, the protoplasts were harvested by centrifugation.

### 5.8. Immunochemical Detection of S-Persulfidated Proteins

S-persulfidated proteins were detected using a modified tag-switch method [23]. The total protein was extracted from rice seedlings with buffer (25 mM of Tris, 100 mM of NaCl, 0.2% Triton X-100, pH 8.0). Blocking buffer consisting of 50 mM of methylsulfonylbenzothiazole that was dissolved in tetrahydrofuran was added to an equal amount of extracted protein solutions and was incubated at 37 °C for 1 h to block free sulfhydryl groups. Proteins were precipitated by acetone to remove the excess and were resuspended in buffer (50 mM of Tris, 2.5% (*w*/*v*) SDS, 20 mM of CN-biotin, pH 8.0) and incubated 3 h at 37 °C. After that, the excess was removed by acetone. The final pellet was resuspended in buffer (50 mM of Tris, 0.5% (*w*/*v*) SDS, pH 8.0). The samples were run on SDS-PAGE and then transferred to a polyvinylidene fluoride membrane. The Western blot was performed with 1:10,000 dilution anti-biotin-HRP (Abcam, Cambridge, MA, USA). Coomassie brilliant blue-stained gels are present to show that equal amounts of proteins were loaded.

### 5.9. Real-Time RT-PCR Analysis

Total RNA was isolated from rice leaves using the Trizol reagent (Invitrogen) according to the manufacturer’s instructions. Real-time quantitative reverse-transcription PCR was performed on a Mastercycler ep^®^ realplex real-time PCR system (Eppendorf, Hamburg, Germany) in a 20 µL PCR amplification using SYBR^®^ Premix Ex Taq™ (TaKaRa, San Jose, CA, USA) according to the manufacturer’s instructions. Related primers and locus numbers of those genes are shown in Supplementary Table S1. The expression level of target genes was presented as x-fold changes relative to the appropriate control experiment after normalized against that of *OsActin1* (*LOC_Os03g50890*) and *OsActin2* (*LOC_Os10g36650*). Each experiment was performed with three replicates (each biological replicate was measured three times).

### 5.10. Statistical Analysis

Statistical analysis was performed using the software of SPSS 17.0. Statistical comparisons were performed by independent samples *t*-test (two-tailed, * *p* < 0.05, ** *p* < 0.01). Multiple comparisons were performed using a one-way ANOVA. Differences were considered significant at *p* < 0.05. All experimental data are presented as mean ± SD.

## Figures and Tables

**Table 1:** The purification and catalytic activity of *OsLCD1* expressed in *E. coli*. The recombinant *His*-tagged *OsLCD1* protein was purified using the Ni-NTA Purification System. CDes and OAS-TL activities were measured as described.

Purification Step	Protein (mg)	Specific Activity (nmol min^−1^ mg^−1^ pro) CDes OAS-TL	Total Activity (nmol min^−1^) CDes OAS-TL	Purification Factor CDes OAS-TL	Yield (%) CDes OAS-TL
Crude extract	1.15	8.02 1.90 × 10^3^	9.22 2.77 × 10^3^	__ __	__ __
Ni-NTA chromatography	0.14	23.93 0.72 × 10^3^	3.35 0.13 × 10^3^	2.98 0.38	36.33 4.69

**Table 2:** Catalytic properties of the recombinant *His*-tagged *OsLCD1* for the CDes and OAS-TL enzymatic reactions. l-Cys was used as a substrate for the DES reaction, whereas OAS and Na_2_S were used as co-substrates for the OAS-TL reaction. Lineweaver–Burk plot was performed to calculate the kinetic constants.

Km (mM Cys)	Vmax (μmol H_2_S min^−1^ mg^−1^ pro)	Km (mM OAS)	Km (mM Na_2_S)	Vmax (μmol l-Cys min^−1^ mg^−1^ pro)
0.15 ± 0.02	0.04 ± 0.01	3.76 ± 0.41	8.13 ± 0.72	1.76 ± 0.32

## Supplementary Materials

The Supplementary Materials are available online at https://www.mdpi.com/article/10.3390/ijms222212119/s1.
