# Supplementary material for: Persulfidation of Nitrate Reductase 2 Is Involved in l-Cysteine Desulfhydrase-Regulated Rice Drought Tolerance
Source: Int J Mol Sci. 2021 Nov 9;22(22):12119. doi: 10.3390/ijms222212119 (PMC8624084; doi:10.3390/ijms222212119)
Supplement: Supplementary file 1 [file ijms-22-12119-s001.zip › ijms-1410631-supplementary.pdf]

Supporting information for

Persulfidation of NITRATE REDUCTASE 2 is involved in L-CYSTEINE DESULFHYDRASE-regulated rice drought tolerance

Heng Zhou[1], Yin Zhou[1], Feng Zhang[1], Wenxue Guan[1], Ye Su[1], Xingxing Yuan[2], Yanjie Xie[1,*]

[1] Laboratory Center of Life Sciences, College of Life Sciences, Nanjing Agricultural University, Nanjing 210095, PR China; hengzhou@njau.edu.cn (H.Z.); 2020116101@njau.edu.cn (Y.Z.); 2018116103@njau.edu.cn (F.Z.); 1346888210@njau.edu.cn (W.G.); 2017116117@njau.edu.cn (Y.S.)

[2] Institute of Industrial Crops, Jiangsu Academy of Agricultural Sciences, Nanjing 210014, China; yxx@jaas.ac.cn (X.Y.)

* Correspondence: yjxie@njau.edu.cn (Y.X.)

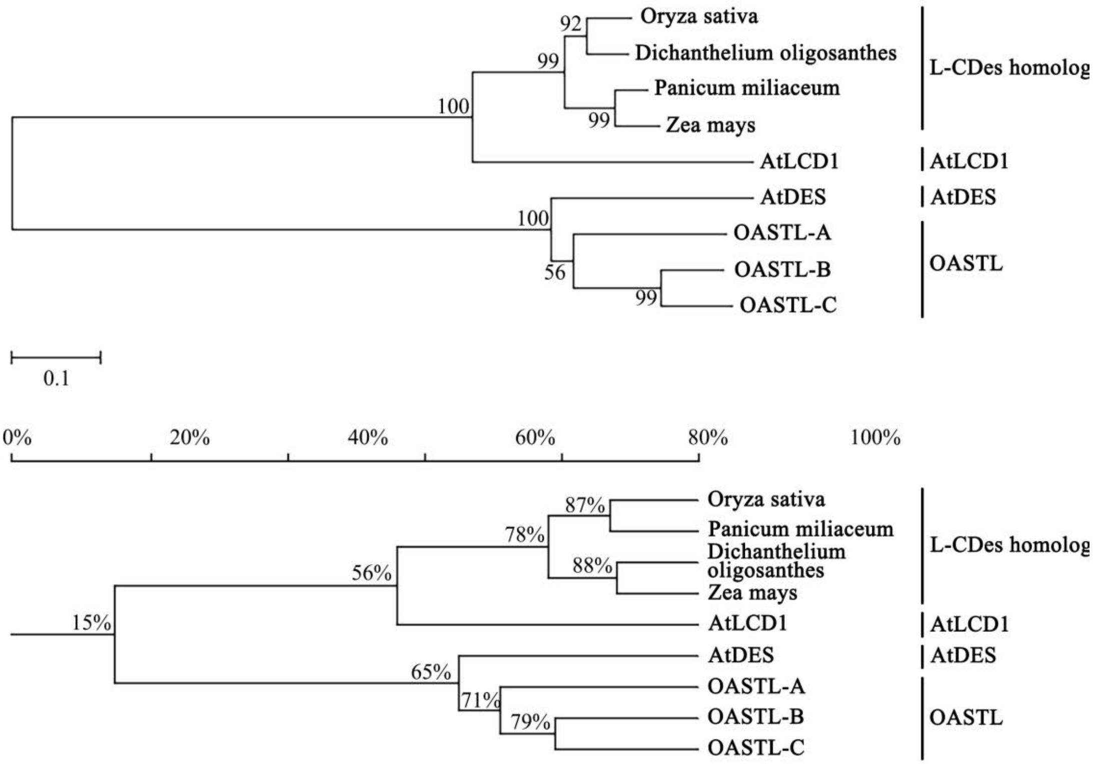

Figure S1. Sequence analysis of CDes, CDes homology and OASTL-A, B, C proteins from plant. Phylogenetic tree analysis of CDes, CDes homology and OAS-TL protein in higher plants. The phylogenetic tree was created by MAGE (Version 6.0) using neighbor-joining method with a bootstrap value of 1000 replicates. Homology tree analysis of CDes, CDes homology and OAS-TL protein in higher plants. The homology tree was created by DNAMAN with default parame.

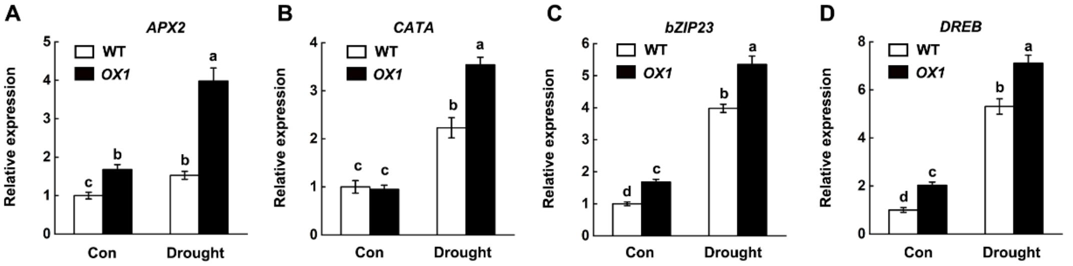

Figure S2. Overexpression of OsLCD1 promoted expression of genes involved in drought stress response. **(A-D)** Relative transcript levels of genes involved in drought stress response in rice seedling leaves were quantified after 4 days of drought stress by qRT-PCR. Expression levels are relative to corresponding untreated wild type samples (control), after normalization to *OsActin1* and *OsActin2*. Data are means ±SE (n = 3). Lower case letters indicate significant differences at P < 0.05 (Duncan's multiple range tests).

Table S1. The primer used in this study.

Target genes	Primer sequence
OsActin1	5'-CAACACCCCTGCTATGTACG-3'
	5'-CATCACCAGAGTCCAACACAA-3'
OsActin2	5'-ACAGGTATTGTGTTGGACTCTGG-3'
	5'-AGTAACCACGCTCCGTCAGG-3'
OsNIA1	5'-CCAATTCTTTCATCGTGTTCT-3'
	5'-CATGCAGCATTTCGTTTCT-3'
OsNIA2	5'-ACTGGTGCTGGTGCTTCTGG-3'
	5'-CGGCTGGGTGTTGAGGGACT-3'
OsCATA	5'-CAACCGCAACGTCGACAACTTCTT-3'
	5'-TTCACCGGCAGCATCAGGTAGTTT-3'
OsAPX2	5'-CATTGCCCGTGGTACTCT-3'
	5'-TTTCATACCAACACATCT-3'
OsbZIP23	5'-GACATGATGTGCTTCGGTG-3'
	5'-GAACTCCTCCAGCGTGATC-3'
OsDREB	5'-GAAACACACACACAAATCCGAA-3'
	5'-GTCTCCCTGAACTTGGTCC-3'
